# Supplementary material for: Deep Residual Learning-Based Classification with Identification of Incorrect Predictions and Quantification of Cellularity and Nuclear Morphological Features in Digital Pathological Images of Common Astrocytic Tumors
Source: Cancers (Basel). 2024 Jul 3;16(13):2449. doi: 10.3390/cancers16132449 (PMC11240501; doi:10.3390/cancers16132449)
Supplement: Supplementary file 1 [file cancers-16-02449-s001.zip › Supplementary Materials.docx]

**Supplementary Materials**

**Table S1.** The details of testing results using ResNet-50 for classifying diffuse astrocytoma, anaplastic astrocytoma, and glioblastoma.

| **Testing Case No.** | **Diagnosis** | **Characteristic Morphological Features** | **Prediction** | | **Inclusion Criterion of 0.00** | | **Inclusion Criterion of 0.02** | | **Inclusion Criterion of 0.05** | |
| --- | --- | --- | --- | --- | --- | --- | --- | --- | --- | --- |
|  |  |  | **Count** | **Ratio** | **Ratio** | **Classification** | **Ratio** | **Classification** | **Ratio** | **Classification** |
| Case 1 | Glioblastoma | Diffuse astrocytoma | 1 | 0.005 | 0.005 | **Glioblastoma** | ~~0.005~~ | **Glioblastoma** | ~~0.005~~ | **Glioblastoma** |
|  |  | Anaplastic astrocytoma | 32 | 0.149 | 0.149 |  | 0.149 |  | 0.149 |  |
|  |  | Glioblastoma tumor cell area | 181 | 0.842 | **0.842** |  | **0.842** |  | **0.842** |  |
|  |  | Glioblastoma necrosis area |  |  |  |  |  |  |  |  |
|  |  | Glioblastoma microvascular proliferation area | 1 | 0.005 | **0.005** |  | ~~0.005~~ |  | ~~0.005~~ |  |
| Case 2 | Glioblastoma | Diffuse astrocytoma |  |  |  | **Glioblastoma** |  | **Glioblastoma** |  | **Glioblastoma** |
|  |  | Anaplastic astrocytoma |  |  |  |  |  |  |  |  |
|  |  | Glioblastoma tumor cell area | 609 | 0.819 | **0.819** |  | **0.819** |  | **0.819** |  |
|  |  | Glioblastoma necrosis area | 135 | 0.181 | **0.181** |  | **0.181** |  | **0.181** |  |
|  |  | Glioblastoma microvascular proliferation area |  |  |  |  |  |  |  |  |
| Case 3 | Glioblastoma | Diffuse astrocytoma |  |  |  | **Glioblastoma** |  | **Glioblastoma** |  | **Glioblastoma** |
|  |  | Anaplastic astrocytoma |  |  |  |  |  |  |  |  |
|  |  | Glioblastoma tumor cell area | 77 | 0.226 | **0.226** |  | **0.226** |  | **0.226** |  |
|  |  | Glioblastoma necrosis area | 264 | 0.774 | **0.774** |  | **0.774** |  | **0.774** |  |
|  |  | Glioblastoma microvascular proliferation area |  |  |  |  |  |  |  |  |
| Case 4 | Diffuse astrocytoma | Diffuse astrocytoma | 11 | 1.000 | **1.000** | **Diffuse astrocytoma** | **1.000** | **Diffuse astrocytoma** | **1.000** | **Diffuse astrocytoma** |
|  |  | Anaplastic astrocytoma |  |  |  |  |  |  |  |  |
|  |  | Glioblastoma tumor cell area |  |  |  |  |  |  |  |  |
|  |  | Glioblastoma necrosis area |  |  |  |  |  |  |  |  |
|  |  | Glioblastoma microvascular proliferation area |  |  |  |  |  |  |  |  |
| Case 5 | Glioblastoma | Diffuse astrocytoma |  |  |  | **Glioblastoma** |  | **Glioblastoma** |  | **Glioblastoma** |
|  |  | Anaplastic astrocytoma | 1 | 0.016 | 0.016 |  | ~~0.016~~ |  | ~~0.016~~ |  |
|  |  | Glioblastoma tumor cell area | 6 | 0.097 | **0.097** |  | **0.097** |  | **0.097** |  |
|  |  | Glioblastoma necrosis area | 55 | 0.887 | **0.887** |  | **0.887** |  | **0.887** |  |
|  |  | Glioblastoma microvascular proliferation area |  |  |  |  |  |  |  |  |
| Case 6 | Glioblastoma | Diffuse astrocytoma |  |  |  | **Glioblastoma** |  | **Glioblastoma** |  | **Glioblastoma** |
|  |  | Anaplastic astrocytoma | 2 | 0.016 | 0.016 |  | ~~0.016~~ |  | ~~0.016~~ |  |
|  |  | Glioblastoma tumor cell area | 123 | 0.961 | **0.961** |  | **0.961** |  | **0.961** |  |
|  |  | Glioblastoma necrosis area | 3 | 0.023 | **0.023** |  | **0.023** |  | ~~0.023~~ |  |
|  |  | Glioblastoma microvascular proliferation area |  |  |  |  |  |  |  |  |
| Case 7 | Glioblastoma | Diffuse astrocytoma |  |  |  | **Glioblastoma** |  | **Glioblastoma** |  | **Glioblastoma** |
|  |  | Anaplastic astrocytoma | 73 | 0.453 | 0.453 |  | 0.453 |  | 0.453 |  |
|  |  | Glioblastoma tumor cell area | 88 | 0.547 | **0.547** |  | **0.547** |  | **0.547** |  |
|  |  | Glioblastoma necrosis area |  |  |  |  |  |  |  |  |
|  |  | Glioblastoma microvascular proliferation area |  |  |  |  |  |  |  |  |
| Case 8 | Glioblastoma | Diffuse astrocytoma |  |  |  | **Glioblastoma** |  | **Glioblastoma** |  | **Glioblastoma** |
|  |  | Anaplastic astrocytoma | 14 | 0.006 | 0.006 |  | ~~0.006~~ |  | ~~0.006~~ |  |
|  |  | Glioblastoma tumor cell area | 2247 | 0.994 | **0.994** |  | **0.994** |  | **0.994** |  |
|  |  | Glioblastoma necrosis area |  |  |  |  |  |  |  |  |
|  |  | Glioblastoma microvascular proliferation area |  |  |  |  |  |  |  |  |
| Case 9 | Glioblastoma | Diffuse astrocytoma |  |  |  | **Glioblastoma** |  | **Glioblastoma** |  | **Glioblastoma** |
|  |  | Anaplastic astrocytoma | 3 | 0.006 | 0.006 |  | ~~0.006~~ |  | ~~0.006~~ |  |
|  |  | Glioblastoma tumor cell area | 375 | 0.725 | **0.725** |  | **0.725** |  | **0.725** |  |
|  |  | Glioblastoma necrosis area | 135 | 0.261 | **0.261** |  | **0.261** |  | **0.261** |  |
|  |  | Glioblastoma microvascular proliferation area | 4 | 0.008 | **0.008** |  | ~~0.008~~ |  | ~~0.008~~ |  |
| Case 10 | Glioblastoma | Diffuse astrocytoma |  |  |  | **Glioblastoma** |  | **Glioblastoma** |  | **Glioblastoma** |
|  |  | Anaplastic astrocytoma |  |  |  |  |  |  |  |  |
|  |  | Glioblastoma tumor cell area | 1 | 0.050 | **0.050** |  | **0.050** |  | **0.050** |  |
|  |  | Glioblastoma necrosis area | 19 | 0.950 | **0.950** |  | **0.950** |  | **0.950** |  |
|  |  | Glioblastoma microvascular proliferation area |  |  |  |  |  |  |  |  |
| Case 11 | Glioblastoma | Diffuse astrocytoma |  |  |  | **Glioblastoma** |  | **Glioblastoma** |  | **Glioblastoma** |
|  |  | Anaplastic astrocytoma | 14 | 0.043 | 0.043 |  | 0.043 |  | ~~0.043~~ |  |
|  |  | Glioblastoma tumor cell area | 251 | 0.775 | **0.775** |  | **0.775** |  | **0.775** |  |
|  |  | Glioblastoma necrosis area | 50 | 0.154 | **0.154** |  | **0.154** |  | **0.154** |  |
|  |  | Glioblastoma microvascular proliferation area | 9 | 0.028 | **0.028** |  | **0.028** |  | ~~0.028~~ |  |
| Case 12 | Glioblastoma | Diffuse astrocytoma |  |  |  | **Glioblastoma** |  | **Glioblastoma** |  | **Glioblastoma** |
|  |  | Anaplastic astrocytoma | 15 | 0.115 | 0.115 |  | 0.115 |  | 0.115 |  |
|  |  | Glioblastoma tumor cell area | 96 | 0.738 | **0.738** |  | **0.738** |  | **0.738** |  |
|  |  | Glioblastoma necrosis area | 19 | 0.146 | **0.146** |  | **0.146** |  | **0.146** |  |
|  |  | Glioblastoma microvascular proliferation area |  |  |  |  |  |  |  |  |
| Case 13 | Diffuse astrocytoma | Diffuse astrocytoma | 5025 | 0.998 | 0.998 | **Glioblastoma** | **0.998** | **Diffuse astrocytoma** | **0.998** | **Diffuse astrocytoma** |
|  |  | Anaplastic astrocytoma | 3 | 0.001 | 0.001 |  | ~~0.001~~ |  | ~~0.001~~ |  |
|  |  | Glioblastoma tumor cell area |  |  |  |  |  |  |  |  |
|  |  | Glioblastoma necrosis area | 9 | 0.002 | **0.002** |  | ~~0.002~~ |  | ~~0.002~~ |  |
|  |  | Glioblastoma microvascular proliferation area |  |  |  |  |  |  |  |  |
| Case 14 | Diffuse astrocytoma | Diffuse astrocytoma | 216 | 0.722 | 0.722 | **Glioblastoma** | 0.722 | **Glioblastoma** | 0.722 | **Glioblastoma** |
|  |  | Anaplastic astrocytoma |  |  |  |  |  |  |  |  |
|  |  | Glioblastoma tumor cell area | 22 | 0.074 | **0.074** |  | **0.074** |  | **0.074** |  |
|  |  | Glioblastoma necrosis area | 60 | 0.201 | **0.201** |  | **0.201** |  | **0.201** |  |
|  |  | Glioblastoma microvascular proliferation area | 1 | 0.003 | **0.003** |  | ~~0.003~~ |  | ~~0.003~~ |  |
| Case 15 | Anaplastic astrocytoma | Diffuse astrocytoma |  |  |  | **Glioblastoma** |  | **Glioblastoma** |  | **Glioblastoma** |
|  |  | Anaplastic astrocytoma |  |  |  |  |  |  |  |  |
|  |  | Glioblastoma tumor cell area | 146 | 1.000 | **1.000** |  | **1.000** |  | **1.000** |  |
|  |  | Glioblastoma necrosis area |  |  |  |  |  |  |  |  |
|  |  | Glioblastoma microvascular proliferation area |  |  |  |  |  |  |  |  |
| Case 16 | Glioblastoma | Diffuse astrocytoma |  |  |  | **Glioblastoma** |  | **Glioblastoma** |  | **Glioblastoma** |
|  |  | Anaplastic astrocytoma |  |  |  |  |  |  |  |  |
|  |  | Glioblastoma tumor cell area | 102 | 0.650 | **0.650** |  | **0.650** |  | **0.650** |  |
|  |  | Glioblastoma necrosis area | 47 | 0.299 | **0.299** |  | **0.299** |  | **0.299** |  |
|  |  | Glioblastoma microvascular proliferation area | 8 | 0.051 | **0.051** |  | **0.051** |  | **0.051** |  |
| Case 17 | Diffuse astrocytoma | Diffuse astrocytoma | 5280 | 0.998 | 0.998 | **Glioblastoma** | **0.998** | **Diffuse astrocytoma** | **0.998** | **Diffuse astrocytoma** |
|  |  | Anaplastic astrocytoma |  |  |  |  |  |  |  |  |
|  |  | Glioblastoma tumor cell area |  |  |  |  |  |  |  |  |
|  |  | Glioblastoma necrosis area | 9 | 0.002 | **0.002** |  | ~~0.002~~ |  | ~~0.002~~ |  |
|  |  | Glioblastoma microvascular proliferation area |  |  |  |  |  |  |  |  |
| Case 18 | Glioblastoma | Diffuse astrocytoma |  |  |  | **Glioblastoma** |  | **Glioblastoma** |  | **Glioblastoma** |
|  |  | Anaplastic astrocytoma | 6 | 0.083 | 0.083 |  | 0.083 |  | 0.083 |  |
|  |  | Glioblastoma tumor cell area | 10 | 0.139 | **0.139** |  | **0.139** |  | **0.139** |  |
|  |  | Glioblastoma necrosis area | 56 | 0.778 | **0.778** |  | **0.778** |  | **0.778** |  |
|  |  | Glioblastoma microvascular proliferation area |  |  |  |  |  |  |  |  |
| Case 19 | Glioblastoma | Diffuse astrocytoma |  |  |  | **Glioblastoma** |  | **Glioblastoma** |  | **Glioblastoma** |
|  |  | Anaplastic astrocytoma |  |  |  |  |  |  |  |  |
|  |  | Glioblastoma tumor cell area | 269 | 0.982 | **0.982** |  | **0.982** |  | **0.982** |  |
|  |  | Glioblastoma necrosis area |  |  |  |  |  |  |  |  |
|  |  | Glioblastoma microvascular proliferation area | 5 | 0.018 | **0.018** |  | ~~0.018~~ |  | ~~0.018~~ |  |
| Case 20 | Glioblastoma | Diffuse astrocytoma | 3 | 0.010 | 0.010 | **Glioblastoma** | ~~0.010~~ | **Glioblastoma** | ~~0.010~~ | **Glioblastoma** |
|  |  | Anaplastic astrocytoma | 4 | 0.013 | 0.013 |  | ~~0.013~~ |  | ~~0.013~~ |  |
|  |  | Glioblastoma tumor cell area | 248 | 0.795 | **0.795** |  | **0.795** |  | **0.795** |  |
|  |  | Glioblastoma necrosis area | 55 | 0.176 | **0.176** |  | **0.176** |  | **0.176** |  |
|  |  | Glioblastoma microvascular proliferation area | 2 | 0.006 | **0.006** |  | ~~0.006~~ |  | ~~0.006~~ |  |
| Case 21 | Glioblastoma | Diffuse astrocytoma |  |  |  | **Glioblastoma** |  | **Glioblastoma** |  | **Glioblastoma** |
|  |  | Anaplastic astrocytoma |  |  |  |  |  |  |  |  |
|  |  | Glioblastoma tumor cell area | 3 | 1.000 | **1.000** |  | **1.000** |  | **1.000** |  |
|  |  | Glioblastoma necrosis area |  |  |  |  |  |  |  |  |
|  |  | Glioblastoma microvascular proliferation area |  |  |  |  |  |  |  |  |
| Case 22 | Diffuse astrocytoma | Diffuse astrocytoma | 530 | 0.994 | 0.994 | **Glioblastoma** | **0.994** | **Diffuse astrocytoma** | **0.994** | **Diffuse astrocytoma** |
|  |  | Anaplastic astrocytoma |  |  |  |  |  |  |  |  |
|  |  | Glioblastoma tumor cell area |  |  |  |  |  |  |  |  |
|  |  | Glioblastoma necrosis area | 1 | 0.002 | **0.002** |  | ~~0.002~~ |  | ~~0.002~~ |  |
|  |  | Glioblastoma microvascular proliferation area | 2 | 0.004 | **0.004** |  | ~~0.004~~ |  | ~~0.004~~ |  |
| Case 23 | Glioblastoma | Diffuse astrocytoma |  |  |  | **Glioblastoma** |  | **Glioblastoma** |  | **Glioblastoma** |
|  |  | Anaplastic astrocytoma |  |  |  |  |  |  |  |  |
|  |  | Glioblastoma tumor cell area | 106 | 1.000 | **1.000** |  | **1.000** |  | **1.000** |  |
|  |  | Glioblastoma necrosis area |  |  |  |  |  |  |  |  |
|  |  | Glioblastoma microvascular proliferation area |  |  |  |  |  |  |  |  |
| Case 24 | Glioblastoma | Diffuse astrocytoma | 4 | 0.007 | 0.007 | **Glioblastoma** | ~~0.007~~ | **Glioblastoma** | ~~0.007~~ | **Glioblastoma** |
|  |  | Anaplastic astrocytoma | 1 | 0.002 | 0.002 |  | ~~0.002~~ |  | ~~0.002~~ |  |
|  |  | Glioblastoma tumor cell area | 540 | 0.929 | **0.929** |  | **0.929** |  | **0.929** |  |
|  |  | Glioblastoma necrosis area | 29 | 0.050 | **0.050** |  | **0.050** |  | ~~0.050~~ |  |
|  |  | Glioblastoma microvascular proliferation area | 7 | 0.012 | **0.012** |  | ~~0.012~~ |  | ~~0.012~~ |  |
| Case 25 | Diffuse astrocytoma | Diffuse astrocytoma | 1 | 0.002 | 0.002 | **Glioblastoma** | ~~0.002~~ | **Glioblastoma** | ~~0.002~~ | **Anaplastic astrocytoma** |
|  |  | Anaplastic astrocytoma | 449 | 0.943 | 0.943 |  | 0.943 |  | **0.943** |  |
|  |  | Glioblastoma tumor cell area | 16 | 0.034 | **0.034** |  | **0.034** |  | ~~0.034~~ |  |
|  |  | Glioblastoma necrosis area | 8 | 0.017 | **0.017** |  | ~~0.017~~ |  | ~~0.017~~ |  |
|  |  | Glioblastoma microvascular proliferation area | 2 | 0.004 | **0.004** |  | ~~0.004~~ |  | ~~0.004~~ |  |
| Case 26 | Glioblastoma | Diffuse astrocytoma |  |  |  | **Glioblastoma** |  | **Glioblastoma** |  | **Glioblastoma** |
|  |  | Anaplastic astrocytoma | 20 | 0.227 | 0.227 |  | 0.227 |  | 0.227 |  |
|  |  | Glioblastoma tumor cell area | 13 | 0.148 | **0.148** |  | **0.148** |  | **0.148** |  |
|  |  | Glioblastoma necrosis area | 42 | 0.477 | **0.477** |  | **0.477** |  | **0.477** |  |
|  |  | Glioblastoma microvascular proliferation area | 13 | 0.148 | **0.148** |  | **0.148** |  | **0.148** |  |
| Case 27 | Anaplastic astrocytoma | Diffuse astrocytoma |  |  |  | **Glioblastoma** |  | **Glioblastoma** |  | **Glioblastoma** |
|  |  | Anaplastic astrocytoma | 28 | 0.113 | 0.113 |  | 0.113 |  | 0.113 |  |
|  |  | Glioblastoma tumor cell area | 220 | 0.887 | **0.887** |  | **0.887** |  | **0.887** |  |
|  |  | Glioblastoma necrosis area |  |  |  |  |  |  |  |  |
|  |  | Glioblastoma microvascular proliferation area |  |  |  |  |  |  |  |  |
| Case 28 | Glioblastoma | Diffuse astrocytoma |  |  |  | **Glioblastoma** |  | **Glioblastoma** |  | **Glioblastoma** |
|  |  | Anaplastic astrocytoma | 125 | 0.096 | 0.096 |  | 0.096 |  | 0.096 |  |
|  |  | Glioblastoma tumor cell area | 1148 | 0.880 | **0.880** |  | **0.880** |  | **0.880** |  |
|  |  | Glioblastoma necrosis area | 18 | 0.014 | **0.014** |  | ~~0.014~~ |  | ~~0.014~~ |  |
|  |  | Glioblastoma microvascular proliferation area | 14 | 0.011 | **0.011** |  | ~~0.011~~ |  | ~~0.011~~ |  |
| Case 29 | Anaplastic astrocytoma | Diffuse astrocytoma |  |  |  | **Glioblastoma** |  | **Glioblastoma** |  | **Glioblastoma** |
|  |  | Anaplastic astrocytoma | 296 | 0.477 | 0.477 |  | 0.477 |  | 0.477 |  |
|  |  | Glioblastoma tumor cell area | 323 | 0.521 | **0.521** |  | **0.521** |  | **0.521** |  |
|  |  | Glioblastoma necrosis area |  |  |  |  |  |  |  |  |
|  |  | Glioblastoma microvascular proliferation area | 1 | 0.002 | **0.002** |  | ~~0.002~~ |  | ~~0.002~~ |  |
| Case 30 | Glioblastoma | Diffuse astrocytoma |  |  |  | **Glioblastoma** |  | **Glioblastoma** |  | **Glioblastoma** |
|  |  | Anaplastic astrocytoma | 1 | 0.002 | 0.002 |  | ~~0.002~~ |  | ~~0.002~~ |  |
|  |  | Glioblastoma tumor cell area | 7 | 0.011 | **0.011** |  | ~~0.011~~ |  | ~~0.011~~ |  |
|  |  | Glioblastoma necrosis area | 656 | 0.985 | **0.985** |  | **0.985** |  | **0.985** |  |
|  |  | Glioblastoma microvascular proliferation area | 2 | 0.003 | **0.003** |  | ~~0.003~~ |  | ~~0.003~~ |  |
| Case 31 | Glioblastoma | Diffuse astrocytoma |  |  |  | **Glioblastoma** |  | **Glioblastoma** |  | **Glioblastoma** |
|  |  | Anaplastic astrocytoma |  |  |  |  |  |  |  |  |
|  |  | Glioblastoma tumor cell area | 234 | 0.903 | **0.903** |  | **0.903** |  | **0.903** |  |
|  |  | Glioblastoma necrosis area | 18 | 0.069 | **0.069** |  | **0.069** |  | **0.069** |  |
|  |  | Glioblastoma microvascular proliferation area | 7 | 0.027 | **0.027** |  | **0.027** |  | ~~0.027~~ |  |
| Case 32 | Glioblastoma | Diffuse astrocytoma |  |  |  | **Glioblastoma** |  | **Glioblastoma** |  | **Glioblastoma** |
|  |  | Anaplastic astrocytoma |  |  |  |  |  |  |  |  |
|  |  | Glioblastoma tumor cell area | 380 | 0.494 | **0.494** |  | **0.494** |  | **0.494** |  |
|  |  | Glioblastoma necrosis area | 367 | 0.477 | **0.477** |  | **0.477** |  | **0.477** |  |
|  |  | Glioblastoma microvascular proliferation area | 22 | 0.029 | **0.029** |  | **0.029** |  | ~~0.029~~ |  |
| Case 33 | Glioblastoma | Diffuse astrocytoma |  |  |  | **Glioblastoma** |  | **Glioblastoma** |  | **Glioblastoma** |
|  |  | Anaplastic astrocytoma | 11 | 0.004 | 0.004 |  | ~~0.004~~ |  | ~~0.004~~ |  |
|  |  | Glioblastoma tumor cell area | 2582 | 0.948 | **0.948** |  | **0.948** |  | **0.948** |  |
|  |  | Glioblastoma necrosis area | 93 | 0.034 | **0.034** |  | **0.034** |  | ~~0.034~~ |  |
|  |  | Glioblastoma microvascular proliferation area | 37 | 0.014 | **0.014** |  | ~~0.014~~ |  | ~~0.014~~ |  |
| Case 34 | Diffuse astrocytoma | Diffuse astrocytoma | 298 | 1.000 | **1.000** | **Diffuse astrocytoma** | **1.000** | **Diffuse astrocytoma** | **1.000** | **Diffuse astrocytoma** |
|  |  | Anaplastic astrocytoma |  |  |  |  |  |  |  |  |
|  |  | Glioblastoma tumor cell area |  |  |  |  |  |  |  |  |
|  |  | Glioblastoma necrosis area |  |  |  |  |  |  |  |  |
|  |  | Glioblastoma microvascular proliferation area |  |  |  |  |  |  |  |  |
| Case 35 | Glioblastoma | Diffuse astrocytoma |  |  |  | **Glioblastoma** |  | **Glioblastoma** |  | **Glioblastoma** |
|  |  | Anaplastic astrocytoma |  |  |  |  |  |  |  |  |
|  |  | Glioblastoma tumor cell area | 922 | 0.964 | **0.964** |  | **0.964** |  | **0.964** |  |
|  |  | Glioblastoma necrosis area | 12 | 0.013 | **0.013** |  | ~~0.013~~ |  | ~~0.013~~ |  |
|  |  | Glioblastoma microvascular proliferation area | 22 | 0.023 | **0.023** |  | **0.023** |  | ~~0.023~~ |  |
| Case 36 | Glioblastoma | Diffuse astrocytoma |  |  |  | **Glioblastoma** |  | **Glioblastoma** |  | **Glioblastoma** |
|  |  | Anaplastic astrocytoma |  |  |  |  |  |  |  |  |
|  |  | Glioblastoma tumor cell area | 85 | 0.431 | **0.431** |  | **0.431** |  | **0.431** |  |
|  |  | Glioblastoma necrosis area | 104 | 0.528 | **0.528** |  | **0.528** |  | **0.528** |  |
|  |  | Glioblastoma microvascular proliferation area | 8 | 0.041 | **0.041** |  | **0.041** |  | ~~0.041~~ |  |
| Case 37 | Glioblastoma | Diffuse astrocytoma | 1 | 0.027 | 0.027 | **Glioblastoma** | 0.027 | **Glioblastoma** | ~~0.027~~ | **Glioblastoma** |
|  |  | Anaplastic astrocytoma | 2 | 0.054 | 0.054 |  | 0.054 |  | 0.054 |  |
|  |  | Glioblastoma tumor cell area | 15 | 0.405 | **0.405** |  | **0.405** |  | **0.405** |  |
|  |  | Glioblastoma necrosis area | 19 | 0.514 | **0.514** |  | **0.514** |  | **0.514** |  |
|  |  | Glioblastoma microvascular proliferation area |  |  |  |  |  |  |  |  |
| Case 38 | Diffuse astrocytoma | Diffuse astrocytoma | 6 | 0.016 | 0.016 | **Glioblastoma** | ~~0.016~~ | **Glioblastoma** | ~~0.016~~ | **Glioblastoma** |
|  |  | Anaplastic astrocytoma |  |  |  |  |  |  |  |  |
|  |  | Glioblastoma tumor cell area |  |  |  |  |  |  |  |  |
|  |  | Glioblastoma necrosis area | 367 | 0.984 | **0.984** |  | **0.984** |  | **0.984** |  |
|  |  | Glioblastoma microvascular proliferation area |  |  |  |  |  |  |  |  |
| Case 39 | Glioblastoma | Diffuse astrocytoma |  |  |  | **Glioblastoma** |  | **Glioblastoma** |  | **Glioblastoma** |
|  |  | Anaplastic astrocytoma | 9 | 0.014 | 0.014 |  | ~~0.014~~ |  | ~~0.014~~ |  |
|  |  | Glioblastoma tumor cell area | 479 | 0.770 | **0.770** |  | **0.770** |  | **0.770** |  |
|  |  | Glioblastoma necrosis area | 71 | 0.114 | **0.114** |  | **0.114** |  | **0.114** |  |
|  |  | Glioblastoma microvascular proliferation area | 63 | 0.101 | **0.101** |  | **0.101** |  | **0.101** |  |
| Inclusion criteria of 0.00 means any patch ratio of the predicted characteristic morphological feature is considered for classification.  The inclusion criteria of 0.02 mean that only when the patch ratio of the predicted characteristic morphological features reaches 0.02, is it considered for classification.  The inclusion criteria of 0.05 mean that only when the patch ratio of the predicted characteristic morphological features reaches 0.05, is it considered for classification.  Number with strikethrough means criteria exclude the patch ratio.  The bold number in the ratio column indicates the determinant(s) for classification.  Red text indicates incorrect classification. | | | | | | | | | | |
